# Root exudation of phytosiderophores from soil-grown wheat

**DOI:** 10.1111/nph.12868

**Published:** 2014-06-02

**Authors:** Eva Oburger, Barbara Gruber, Yvonne Schindlegger, Walter D C Schenkeveld, Stephan Hann, Stephan M Kraemer, Walter W Wenzel, Markus Puschenreiter

**Affiliations:** 1 Department of Forest and Soil Sciences, University of Natural Resources and Life Sciences ViennaKonrad-Lorenz Straße 24, A-3430, Tulln, Austria; 2 Department of Chemistry, University of Natural Resources and Life Sciences ViennaMuthgasse 18, A-1190, Vienna, Austria; 3 Department of Environmental Geosciences, University of ViennaAlthanstraße 14, UZAII, A-1090, Vienna, Austria

**Keywords:** 2′-deoxymugineic acid (DMA), iron deficiency, phytosiderophore, rhizosphere, strategy II, trace elements, *Triticum aestivum* cv Tamaro

## Abstract

- For the first time, phytosiderophore (PS) release of wheat (*Triticum aestivum* cv Tamaro) grown on a calcareous soil was repeatedly and nondestructively sampled using rhizoboxes combined with a recently developed root exudate collecting tool. As in nutrient solution culture, we observed a distinct diurnal release rhythm; however, the measured PS efflux was *c*. 50 times lower than PS exudation from the same cultivar grown in zero iron (Fe)-hydroponic culture.
- Phytosiderophore rhizosphere soil solution concentrations and PS release of the Tamaro cultivar were soil-dependent, suggesting complex interactions of soil characteristics (salinity, trace metal availability) and the physiological status of the plant and the related regulation (amount and timing) of PS release.
- Our results demonstrate that carbon and energy investment into Fe acquisition under natural growth conditions is significantly smaller than previously derived from zero Fe-hydroponic studies. Based on experimental data, we calculated that during the investigated period (21–47 d after germination), PS release initially exceeded Fe plant uptake 10-fold, but significantly declined after *c*. 5 wk after germination.
- Phytosiderophore exudation observed under natural growth conditions is a prerequisite for a more accurate and realistic assessment of Fe mobilization processes in the rhizosphere using both experimental and modeling approaches.

## Introduction

The release of phytosiderophores (PSs) by grass species is considered a highly efficient iron (Fe) acquisition mechanism. These low-molecular-weight, nonproteinogenic amino acids form soluble complexes with Fe(III) that are taken up as the intact PS–metal complex (strategy II), with Fe remaining in its oxidized form, Fe(III). Other micronutrients, such as copper (Cu), zinc (Zn), nickel (Ni) and cobalt (Co), have also been found to form stable complexes with PS and be taken up as a PS complex; however, transport up-regulation has so far only be confirmed under Fe deficiency (Römheld & Marschner, 1990; Schaaf *et al*., 2004). Overexpression of genes involved in PS release has been demonstrated upon Fe deficiency (Nozoye *et al*., 2011), but the effect of an insufficient supply of other micronutrients has not yet been investigated on a molecular level.

The dynamics of PS release appear to be well investigated; however, the vast majority of studies were carried out in nutrient solution culture as this facilitates both exudate sampling and analysis (e.g. see references included in Table 1). By placing an agar sheet on Fe-starved, solution-grown barley roots (*Hordeum vulgare* Europa), Marschner *et al*. (1987) showed that PS release was *c*. two to five times higher in the apical than in the basal root zone and demonstrated the same trend for ^59^Fe-PS uptake. Furthermore, increased PS exudation and ^59^Fe-PS uptake were also observed in the zone of lateral root formation (*c*. 1.5 times higher than at the root apices). When growing under Fe-deficient conditions, PS release by the majority of investigated graminaceous species has been found to follow a distinct diurnal rhythm (e.g. *Hordeum vulgare* (Takagi *et al*., 1984), *Triticum aestivum* (Zhang *et al*., 1991; Reichman & Parker, 2007), *Hordelymus europaeus* (Gries & Runge, 1992), and *Festuca rubra* (Ma *et al*., 2003), *Lolium perenne* and *Poa pratensis* (Ueno & Ma, 2009), with the highest concentrations being exuded between *c*. 2–6 h after the onset of light. It has been further shown that this exudation pattern optimizes iron mobilization in the rhizosphere (Reichard *et al*., 2007). The exudation peak timing appears to differ between species (Ueno & Ma, 2009), and for the majority of graminaceous species, such as maize or rice, direct evidence for diurnal PS exudation dynamics is still missing (Yehuda *et al*., 1996; Inouye *et al*., 2006).

**Table 1:** Comparison of phytosiderophore (PS) exudation rates of different species and cultivars grown in nutrient solution culture

Reference	Species/cultivar	Hydroponic growth conditions	PS sampling/analysis	PS exudation rates (pmol g^−1^ DW s^−1^)		
Clark *et al*. (1988)			24 h/indirectly ^59^Fe(OH)_3_	**11 DAG**	**15 DAG**	**19 DAG**
	*Sorghum bicolor*, MS 35-1	−Fe		1343	64	35
	*Sorghum bicolor*, SPV-393	−Fe		2569	35	213
	*Sorghum bicolor*, Sudangrass3	−Fe		7338	125	160
	*Hordeum vulgare* Europa	−Fe		5775	2697	3079
		+Fe		*9*	118	557
Von Wiren *et al*. (1995)			2 h/HPLC analysis*		**32 DAG**	**35 DAG**
	*Hordeum vulgare*, Taiga	−Fe				∼ 1030
		+Fe				∼ 6
	*Sorghum bicolor*, Y-303 A	−Fe			∼ 150	
		+Fe			∼ 1	
Cakmak *et al*. (1994)			4 h/HPLC analysis*		**15 DAG**	**25 DAG**
	*Triticum aestivum*, Aroona	−Fe			∼ 1500	∼ 1100
		−Fe, Zn			∼ 2300	∼ 850
		−Zn			∼ 460	∼ 30
			+Fe		∼ 70	∼ 20
Schindlegger *et al*. (2014)			4 h/LC-MS/MS analysis			**28 DAG**
	*Triticum aestivum*, Tamaro	−Fe				2200
Gries *et al*. (1998)					**13 DAG**	
	*Hordelymus europaeus*	−Fe	2 h/HPLC analysis		2500	
		−Cu			1007	
		−Zn			208	
		−Mn			361	
		Control			361	
Yehuda *et al*. (1996)			2 h/HPLC analysis*		**15 DAG**	
	*Zea mais*, Alice	−Fe			∼ 800	

Exudation rates were converted to SI units to facilitate comparison.

* Data retrieved from figures. Bold numbers represent the exudate sampling time point expressed in d after germination (DAG).

HPLC, high-performance liquid chromatography.

Several hydroponic growth studies also showed that PS release decreases with increasing Fe availability (Takagi *et al*., 1984; Crowley *et al*., 2002) and differs not only between species but also between different genotypes and plant age, with PS exudation rates (per unit dry mass) generally decreasing with plant age (Table 1 and references therein). It is acknowledged that increased PS release also enhances the uptake of Zn and Cu (Zhang *et al*., 1991; Von Wiren *et al*., 1996; Gries *et al*., 1998), but the initiation of PS release by other micronutrient deficiencies is less well understood.

Hydroponic growth techniques are a powerful tool to investigate plant responses to environmental stresses such as nutrient deficiency or heavy metal toxicity, as conditions can be controlled and manipulated as needed. Nevertheless, growth and/or exudate sampling conditions differ significantly from a natural soil environment, not only in terms of nutrient availability, but also with regard to O_2_/CO_2_ concentration, microbial community composition and mechanical impedance, with the latter particularly affecting root morphology and anatomy (e.g. root hair and Casparian band development), potentially resulting in different responses to nutrient deficiency or heavy metal toxicity. Considering the sensitivity of Fe nutritional status and PS release to environmental conditions (in particular soluble Fe availability; e.g. Crowley *et al*., 2002), simple extrapolations from exudation rates obtained in nutrient solution culture to soil conditions should be handled with care. Fe deficiency in soil-grown plants is mainly found on alkaline soils, rich in calcium carbonate, where Fe phytoavailability is limited by the pH-dependent low solubility of Fe. However, zero-Fe concentrations as obtained in nutrient solution culture will never occur and feedbacks between iron availability and plant iron acquisition strategies may exist. Therefore, PS release rates in soil can be expected to be lower than those observed under hydroponic conditions.

Investigating root exudation in soil is highly challenging owing to the complexity and inaccessibility of the root system. Additionally, adsorption to the soil matrix as well as microbial degradation, but also analytical challenges as a result of the low sample concentrations, further aggravates the determination of PS release rates into soil solution. Consequently, there are only a few studies looking at PS concentrations in soil solution and release from soil-grown plants. So far, neither PS soil solution concentration nor directly sampled release rates from soil-grown plants have been reported. Shi *et al*. (1988) used a two-stage growth approach to investigate PS concentrations in a calcareous soil by pregrowing barley seedlings placed in a nylon mesh compartment under hydroponic conditions and thereafter exposing the chlorotic plants to a soil compartment for 7 or 9 d. This simple approach enabled the authors to sample and extract the rhizosphere soil for PSs using 10% (NH_4_)_2_CO_3_. Accounting for the PS extraction efficiency and assuming a natural soil water content of 15–20%, the authors concluded that the PS concentration in the rhizosphere can be expected to be in the 10^−6^ M range. By deploying an agar sheet on 3-wk-old barley roots (*Hordeum vulgare* L. cv Europa), Römheld (1991) showed that PS release rates of barley grown in a calcareous soil (63% CaCO_3_) were *c*. five times higher than those of barley plants grown in the same soil but with 0.3% Fe-citrate solution applied to their leaves, but this approach only allowed an intrastudy comparison and no DW-based exudation rates could be deduced. To the best of our knowledge, these are the only studies investigating PS dynamics in soil.

Here, we aim to quantify PS exudation rates from soil-grown wheat (*Triticum aestivum* cv Tamaro) directly and to determine PS concentrations in the rhizosphere soil solution *in situ* using well established (microsuction cups (MSCs)/hydroponic exudate sampling) and novel rhizosphere research tools (i.e. recently introduced root exudate collector; Oburger *et al*., 2013). A novel LC-MS/MS based method was employed for 2′-deoxymugineic acid (DMA) quantification (Schindlegger *et al*., 2014), which is the major PS released by wheat (Römheld & Marschner, 1990). Owing to the demonstrated sensitivity of Fe supply on Fe nutritional status and PS release (Crowley *et al*., 2002), we hypothesize that DMA release rates of soil-grown plants will be significantly lower than exudation rates observed for plants grown in zero-Fe nutrient solutions.

## Materials and Methods

### Experimental soils and plant growth conditions

Experimental soils were collected from sites located in Austria (Lassee (Lass), Siebenlinden (SL)), Spain (Santomera (Sant), Xeraco topsoil (Xer T), Xeraco subsoil (Xer L), Italy (Bologna (Bol)) and Saudi Arabia (Nadec (Nad) and named after their geographic origin. The soils from Spain, Italy and Saudi Arabia have been previously used in Fe-deficiency studies (Schenkeveld *et al*., 2008, 2010). All soils but Siebenlinden are highly calcareous and were chosen according to their low but varying diethylenetriamene pentaacetic acid (DTPA)-extractable Fe concentration (determined after Loeppert & Inskeep, 1996). The acidic Siebenlinden soil (pH 4.7) was limed with 10% (w/w) CaCO_3_ (Fluka Analytics) to test the effect of liming on Fe and PS dynamics. The limed soil (SL 10%) was subjected to three wetting (50% of maximum water-holding capacity (MWHC)) and drying (30°C) cycles within 4 wk before plant growth and soil analysis. General soil properties are shown in Table 2. For the plant growth experiments, all soils were fertilized with (mg kg^−1^): NH_4_NO_3_ (530), K_2_HPO_4_ (720), MgSO_4_.7H_2_O (410), H_3_BO_4_ (4), (NH_4_)_6_Mo_7_O_24_.4H_2_O (0.8) and allowed to equilibrate for 1 wk at 60% of the MWHC before planting. All plant experiments were conducted in the glasshouse with an average day : night temperature of 27 : 20°C and a 16 h photoperiod at 400 μmol m^−2^ s^−1^(photosynthetically active radiation).

**Table 2:** General soil parameters of the experimental soils: pH, electric conductivity (EC), maximum water-holding capacity (MWHC), calcium carbonate content (CaCO_3_), soil organic carbon (SOC), and diethylenetriamene pentaacetic acid (DTPA)-extractable trace metal concentrations

							DTPA-extractable		
							
	pH	EC*	MWHC	CaCO_3_	SOC	Clay	Fe	Cu	Zn
	
	CaCl_2_	μS cm^−1^	g kg^−1^	g kg^−1^	g kg^−1^	mg kg^−1^	mg kg^−1^	mg kg^−1^	mg kg^−1^
Santomera	7.8	107	504	499	7.3	306	4.9	1.6	0.5
Bologna	7.6	184	609	150	9.1	268	15.5	3.3	0.5
Xeraco L	7.7	139	615	147	14.2	442	7.5	3.1	5.7
Xeraco T	7.5	470	499	415	28.5	169	75.7	1.4	6.6
Lassee	7.7	154	536	138	15.5	268	4.8	1.2	0.9
Nadec	7.6	1452	327	152	8.3	97	9.6	0.4	0.6
SL +10%	7.6	196	403	80	13.8	103	18.6	0.4	0.4
SL	4.7	107	527	9	13.2	103	47.3	0.1	1.3

* Determined in a 1 : 10 (soil : solution) water extract.

### Expt 1(a): Repetitive and nondestructive sampling of DMA exudation rates from soil-grown wheat – a rhizobox experiment equipped with a root exudate collecting (REC) tool

Wheat was grown in rhizoboxes equipped with a recently developed REC tool described in Oburger *et al*. (2013) (Supporting Information, Fig. S1). Only the highly calcareous Santomera soil was used in this study. Before the plant experiment, stability, circulation recovery and system recovery of DMA were tested as described in Oburger *et al*. (2013). Briefly, for the stability and circulation recovery test, a 70 and a 700 nM DMA solution containing 0.01 g l^−1^ Micropur classic (Katadyn®, Switzerland) was circulated through the exudate collector system for 8 h with no plant or soil contact, and the DMA concentration was monitored at the beginning and the end. The system capture efficiency test was carried out using rhizosphere compartments filled with autoclaved soil (Santomera) and glass fiber wicks as artificial roots that were connected with either a ^14^C-glucose or ^14^C-citrate solution. System capture efficiency was calculated as the percentage recovered in the collector sample based on the total ^14^C activity ‘released’ by the artificial root (i.e. activity in the collector sample plus activity measured in the soil).

In contrast to the setup described in Wenzel *et al*. (2001) where a pregrowth compartment was used, 5-d-old wheat seedlings (10 per rhizobox) were directly placed on the 30 μm nylon membrane that separates the roots from the rhizosphere soil compartment but that allows water, nutrient and metabolite exchange (Fig. S1). This setup was chosen to obtain a sufficiently developed root mat for exudate sampling at an earlier stage of plant development. Five planted rhizoboxes and one unplanted control were set up for a total growth period of 7 wk under the conditions described earlier. To determine DMA exudation rates from soil-grown wheat, a vacuum-tight membrane plate connected with a sampling solution reservoir and sampling vials was attached to the root mat in the rhizoboxes (REC, Fig. S1). Rinsing of the membrane plate was initiated by an electronic circuit unit every 10 min by inducing a vacuum (1 mbar) in the closed exudate collecting system. To investigate plant age-dependent DMA release dynamics, samples were collected 21, 35, 41, and 47 d after germination (DAG). Starting at the onset of light, the roots in the rhizoboxes were repeatedly rinsed by the REC tool for 8 h (later referred to as ‘8 h sampling’) with deionized water containing 0.01 g l^−1^ Micropur. In a previous study, we showed that PS release was not affected by the applied Micropur concentration (Schindlegger *et al*., 2014). To investigate the diurnal rhythm of DMA exudation of soil-grown plants, exudates were sampled 33 and 46 DAG by collecting samples every 2 h during the 12 h day sampling period; the remaining 12 h evening/night period was sampled as one cumulative sample (‘24 h sampling’). After retrieving the samples from the collecting tool, an internal custom-made ^13^C DMA standard (0.5 ml 10 μM ^13^C DMA in 10% formic acid) was immediately added to each sample and samples were frozen (−20°C), freeze-dried (−50°C; 0.06 mbar, Christ Alpha 1-2 LO) and resuspended in 1 ml LC-MS Chromasolv® water (Fluka, Sigma-Aldrich GmbH, Germany) before DMA analysis, which was carried out as described later. After the last exudate sampling (47 DAG), wheat plants were harvested, dried (60°C) and acid-digested with HNO_3_/H_2_O_2_ (Pöykiö *et al*., 2000). Micronutrient concentrations in the root and shoot digests and MSC samples were analyzed by inductively coupled plasma mass spectrometry (ICP-MS; Perkin Elmer, Elan DRCe 9000, Waltham, MA, USA).

### Expt 1(b): Root growth and plant Fe uptake of wheat grown in rhizoboxes

To correctly account for root growth and Fe uptake throughout the root exudate sampling period (21–47 DAG), 15 separate rhizoboxes were prepared as described above but without the REC tool and a set of 3 was harvested after 21, 33, 35, 41 and 47 DAG. During the growth period the SPAD index was regularly monitored using a Minolta SPAD 502 chlorophyll meter, taking 6 readings of random individual leaves per rhizobox. Root and shoot biomass and trace metal concentrations of each rhizobox was determined as described above. The relative Fe accumulation by wheat (roots and shoots) for the different time points was calculated as percentage of total Fe accumulated at the last harvest (47 DAG) and then used to estimate Fe accumulated in wheat plants in the rhizobox-REC Expt 1(a) over time.

### Expt 2(a): DMA and total C release from wheat grown on different soils – a pot experiment with single point hydroponic exudate sampling after careful root washing before harvest

To investigate the effect of different soils on wheat Fe status and DMA release, wheat (*Triticum aestivum* cv Tamaro) was grown on the six calcareous soils (Sant, Bol, Xer T, Xer L, Lass, Nad) and the limed acidic soil (SL 10%) in a simple pot setup. Acid-washed pots (500 ml) were filled with 485 g (DW) of soil, each in three replicates, and wheat seedlings were planted and grown in the glasshouse as described earlier. The water content was kept at 60 ± 10% of the MWHC of each soil individually by weighing throughout the experiment. Exuded DMA was sampled hydroponically at 44 DAG, for 4 h, starting 2 h after the onset of light. Intact roots were carefully separated from soil by continuous, gentle rinsing of the rooted soil block with deionized water until the roots were liberated from mineral particles (visibly clean). To capture exudate compounds and root debris released from damaged cells, roots were submerged two times for 10 min into 200 ml of deionized water containing 0.01 g l^−1^ Micropur classic (Katadyn®). After the washing procedures, roots were then placed in the final sampling solution (150 ml deionized water, 0.01 g l^−1^ Micropur) and kept under glasshouse conditions for the entire sampling period (4 h). Thereafter, the sampling solution was filtered (0.45 μm, nylon, Whatman™ GD/X; GE Healthcare Europe, Freiburg, Germany) and a 10 ml aliquot was used for total dissolved organic C (DOC) analysis (Vario TOC, Elementar, Hanau, Germany). The remaining sample volumes were spiked with an internal ^13^C-DMA standard (0.5 ml 10 μM ^13^C DMA in 10% formic acid), frozen at −20°C and freeze-dried (−50°C; 0.06 mbar) and analyzed as described later. Wheat plants were harvested, dried, digested and analyzed as already described.

### Expt 2(b): DMA and trace metal concentrations in the rhizosphere soil solution – a pot experiment equipped with MSCs

The pot experiment was repeated as described earlier, but to investigate *in situ* DMA rhizosphere concentration, each pot was equipped with a micro-MSC rhizon sampler (5 cm porous hydrophilic membrane (0.12–0.17 μm) composed of a blend of polyvinylpyrrolidine and polyethersulfone with 2.5 mm outer diameter; Rhizosphere Research Products, Wageningen, the Netherlands) and one unplanted control for each soil was included. Additionally, one unplanted control was included for each soil and MSC samples were taken at 42 and 54 DAG from planted and unplanted pots by inducing a vacuum using acid-washed 10 ml syringes. Visual observation indicated that, after 42 DAG, the entire soil of the planted pots was densely rooted and could be considered rhizosphere soil. As microbial degradation is a key process aggravating PS sampling in soil, we tried to minimize microbial activity, by watering the pots the day before MSC sampling with a Bronopol (Thermo Fisher Scientific, Waltham, MA, USA) solution, resulting in a final concentration of 0.2 mg Bronopol g^−1^ soil, a concentration which has been shown to significantly reduce microbial respiration in soil (Rousk *et al*., 2009a). The effective soil solution concentration of Bronopol therefore ranged between 0.5 and 1 g l^−1^ depending on the soils' MWHC. In a preliminary hydroponic experiment, we did not observe a statistically significant decline in PS release by wheat (*T. aestivum* cv Tamaro) up to a Bronopol concentration of 8 g l^−1^ (Schindlegger *et al*., 2014). Therefore no Bronopol-driven artefacts on DMA release were expected.

The MSC sampling was started 4 h after the onset of light and the first few drops of soil solution were discarded. To immediately rule out microbial degradation in the collected solution, 4 μl of NaN_3_ (200 g l^−1^) were pipetted into the syringes before soil solution sampling. Additionally, the syringes were wrapped with aluminum foil to avoid any effects of light. The average soil solution volume sampled ranged between 4 and 6 ml, with 2 ml being immediately frozen at –20°C for DMA (see later) and the remaining sample being acidified with HNO_3_ to a final acid concentration of 1% for trace metal analysis by ICP-MS (Perkin Elmer, Elan DRCe 9000).

### LC- electrospray ionization (ESI)-MS/MS analysis of DMA

Quantification of DMA in soil solution and root exudate samples was performed via LC-ESI-MS/MS (Agilent 1200SL with Agilent 6410 Triple Quadrupole system; Agilent, Waldbronn, Germany). For chromatographic separation, a porous graphitic carbon stationary phase (150 × 2.1 mm Hypercarb® with 3 μm particle size; ThermoFisher Scientific, Vienna, Austria) was run in gradient elution mode (Eluent A, 98% v/v H_2_O, 1% v/v formic acid, 1% v/v methanol; and Eluent B, 98% v/v methanol, 1% v/v formic acid, 1% v/v H_2_O). The gradient program was 0–6 min from 100% to 20% Eluent A, followed by re-equilibration to starting conditions at 6.10 min. The column compartment was thermostated at 60°C. Total analysis time was 14 min and the injection volume was 5 μl. Sample dilution in 1% formic acid assured quantitative dissociation of the metal–DMA complexes and free DMA ligand stabilization. The use of specific MS/MS transitions in multiple reactions monitoring mode allowed background noise reduction, especially from coeluting matrix components, as well as the improvement of signal-to-noise ratio. Quantification was performed within a calibration range of 0.1–80 μM. For internal standardization an in-house synthesized ^13^C_4_-DMA standard (C. Stanetty *et al*., unpublished) at 3.33 μM final concentration was added. The analytical method development and procedure are described and discussed in more detail in (Schindlegger *et al*., 2014).

### Data analysis

Exudation rates were calculated based on root DW. As root biomass was only sampled at the end of the rhizobox-REC experiment (Expt 1a), root biomass used for exudation rate calculation was corrected using the experimentally observed linear growth rate of wheat grown in the same experimental setup (Expt 1b; see also Fig. S1). All statistical analysis was carried out using SPSS 15.0 (SPSS Inc., Chicago, IL, USA). Soluble trace metal concentrations in bulk and rhizosphere were compared using an independent *t*-test (*P* < 0.05). Statistical differences of wheat growth performance on the seven experimental soils and DMA release were determined using a one-way ANOVA in combination with a Student–Newman–Keuls post hoc test (*P* < 0.05). Dependencies between different parameters investigated in the seven soil experiment (Expt 2a) were tested using a bivariate correlation analysis.

## Results

### Expt 1(a): DMA exudation rate dynamics from soil-grown plants – REC experiment

**Fig. 1:**
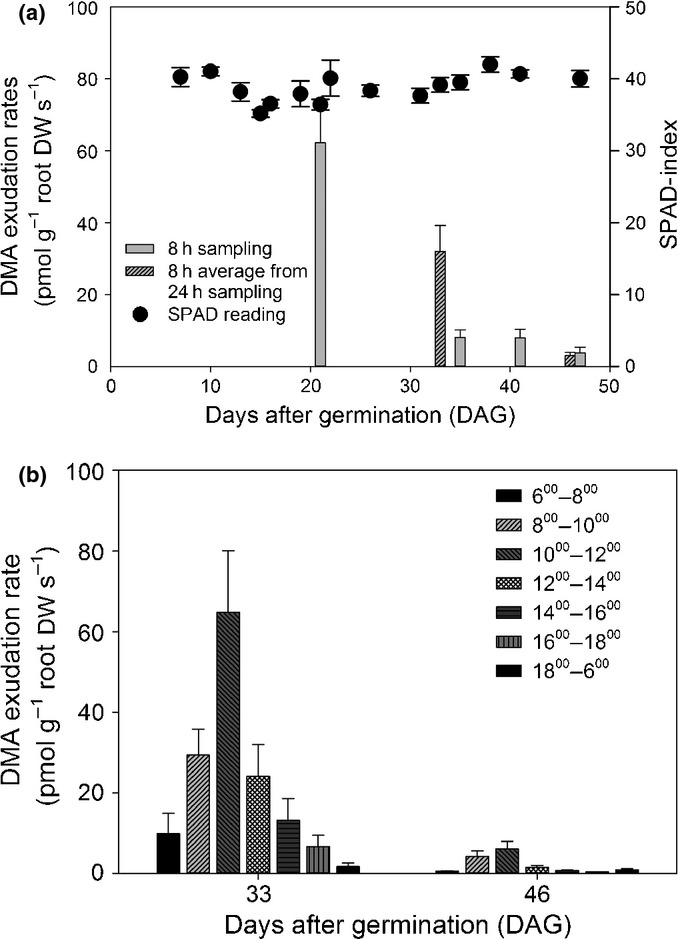
2′-Deoxymugineic acid (DMA) exudation rates of wheat (*Triticum aestivum* cv Tamaro) grown in rhizoboxes filled with calcareous soil (Santomera) and sampled by the root exudate collecting (REC) tool. (a) Plant age-dependent exudation rates sampled for an 8 h period starting at the onset of light (left axis) and concurrent changes in the SPAD index (right axis) (b) Plant age-dependent diurnal rhythm of DMA release. Values represent means ± SE (DMA, *n* = 5; SPAD, *n* = 18).

Stability and circulation recovery tests of the REC tool showed a recovery of 98 ± 4% for both DMA concentrations circulating through the REC tool in the absence of soil (data not shown). System recovery tests in the presence of the experimental soil using radioactive tracers (^14^C-labeled glucose and citrate) revealed an exudate capture efficiency of 77% for both compounds (data not shown). This experimentally determined capture efficiency was used to correct measured DMA concentrations, assuming that the interactions between DMA and soil are similar. Results from repetitive root exudate sampling of wheat grown in the highly calcareous Santomera soil are shown in Fig. 1(a,b). Wheat plants showed signs of chlorosis during the entire experimental period (21–47 DAG), showing leaf tip necrosis and single yellowish leaves (SPAD values of 10–27) while for the majority of leaves measured SPAD indices ranged between 35 and 45 (Fig. 1a). SPAD readings (Fig. 1) showed a slight decline at 13 DAG, followed by a period of fluctuation (at 16–33 DAG) and remained fairly constant towards the end of the investigated period (for statistical results, see Table S1). Both sampling regimes (8 and 24 h stepwise sampling) revealed that DMA release rates (g^–1^ root DW) significantly declined with increasing plant age. DMA exudation rates measured during the 8 h peak release period ranged from 62 ± 11 to 3 ± 1 pmol DMA g^−1^ root DW s^−1^, with DMA release being *c*. 20 times higher on the first sampling day (21 DAG) than on the last (47 DAG) (Fig. 1a). Stepwise sampling over 24 h showed a distinct diurnal rhythm, with DMA exudation peaking between 4 and 6 h after the onset of light (Fig. 1b).

### Expt 1(b): DMA and total C release from wheat grown on different soils

**Fig. 2:**
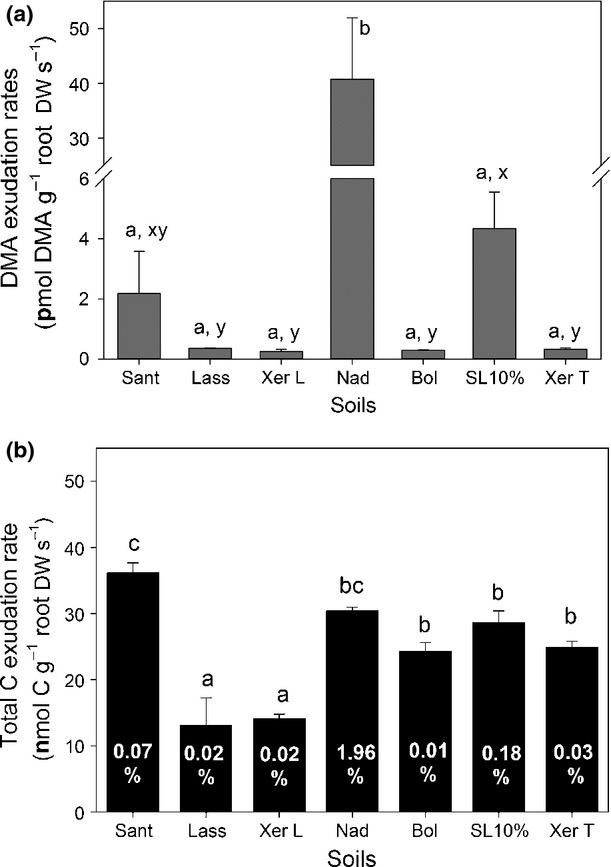
2′-Deoxymugineic acid (DMA) exudation rates (a) and total carbon (C) exudation rates (b) (nmol C g^−1^ root DW s^−1^, derived from total dissolved organic C analyzed) of wheat (*Triticum aestivum* cv Tamaro) grown on seven different calcareous soils. White numbers represent DMA-derived C as a percentage of total C released. Exudation was sampled hydroponically at 44 d after germination (DAG). Values represent means ± SE (*n* = 3). Letters indicate significant differences of DMA and total C release, with *P* < 0.05. Letters a, b: ANOVA including all soils, letters x, y: ANOVA excluding the saline soil Nadec. Sant, Santomera; Xer T, Xeraco topsoil; Xer L, Xeraco subsoil; Bol, Bologna; Nad, Nadec; Lass, Lassee; SL 10%, Siebenlinden + 10% CaCO_3_.

The collection of root exudates in hydroponic solution after careful separation of the roots from the soil revealed significant differences in DMA release rates of wheat when grown on different soils. After 44 d of growth, DMA exudation rates ranged from 0.2 to 41 pmol DMA g^−1^ root DW s^−1^ (Fig. 2a). Interestingly, by far the highest DMA release was observed in a saline soil (Nadec, DTPA-Fe, 9.6 mg kg^−1^), followed by the limed acidic soil (SL 10%, DTPA-Fe, 14 mg kg^−1^) and a highly calcareous soil (Santomera, DTPA-Fe, 4.9 mg kg^−1^). Even though differences in chemical Fe availability (DTPA-extractable Fe, Table 2) for the soils Lassee, Xeraco L, Bologna, and Xeraco T would suggest differently, DMA release rates of wheat grown on the other four experimental soils were in a similar range (0.2–0.4 pmol DMA g^−1^ root DW s^−1^; Fig. 2a). Despite visible chlorosis symptoms, Fe shoot concentrations also differed, but were all around or above the average tissue concentration required for adequate growth, while Zn and Cu tissue concentrations indicated insufficient phytoavailability of these trace metals in some of the experimental soils, particularly in the saline (Nadec) and limed soil (SL + 10%; Table 3). Statistical correlation analysis both including (Table S2A) and excluding the saline soil (Table S2B) revealed that DMA release rates were negatively correlated with shoot Zn. When including the saline soil in the data analysis, a negative correlation for Cu was also observed, while excluding Nadec resulted in a positive correlation of DMA exudation with shoot Fe. Total carbon release of wheat also differed between the soils, but showed a different pattern than that observed for DMA. Release rates ranged from 13 (Lassee) to 36 (Santomera) nmol C g^−1^ root DW s^−1^ (derived from DOC, Fig. 2b). Depending on the soil, DMA-derived C accounted for 0.01 up to 2% of total C released. The highest contribution of DMA-derived C to total C released was observed for wheat plants grown in the saline soil.

**Table 3:** Plant (wheat, *Triticum aestivum* cv Tamaro) biomass and iron (Fe), zinc (Zn) and copper (Cu) shoot tissue concentration after 44 d after germination (Expt 2a)

	Shoot	Root	Shoot Fe	Shoot Zn	
				
Soil	DW (g)	DW (g)		mg kg^−1^	Shoot Cu
Santomera	2.6 ± 0.2 c,d	1.4 ± 0.3 b	267 ± 10 a,b	22 ± 1 b,c	9.1 ± 0.5 a
Bologna	2.4 ± 0.1 d	1.7 ± 0.1 a,b	213 ± 38 b,c	22 ± 0 b,c	6.0 ± 0.2 b,c
Xeraco L	3.2 ± 0.0 a,b	3.4 ± 0.3 a	182 ± 25 b,c	31 ± 1 a	7.1 ± 0.2 b
Xeraco T	3.5 ± 0.0 a	1.9 ± 0.1 a,b	112 ± 17 c	25 ± 0 b	6.1 ± 0.2 b,c
Lassee	3.1 ± 0.1 a,b	2.2 ± 0.9 a,b	197 ± 28 b,c	23 ± 3 b,c	6.5 ± 0.5 b
Nadec	2.5 ± 0.2 c,d	0.8 ± 0.1 b	112 ± 10 c	12 ± 0 d	4.0 ± 0.1 d
SL+10%	3.0 ± 0.1 b,c	1.7 ± 0.2 a,b	327 ± 18 a	17 ± 3 c	5.2 ± 0.2 c
Critical range*			50–150	15–20	1–5

Values represent means + SE (*n =* 3). Letters indicate significant differences (*P* < 0.05).

* Broadley *et al*. (2012).

### Expt 2(b): DMA and trace metal concentrations in the rhizosphere soil solution

**Fig. 3:**
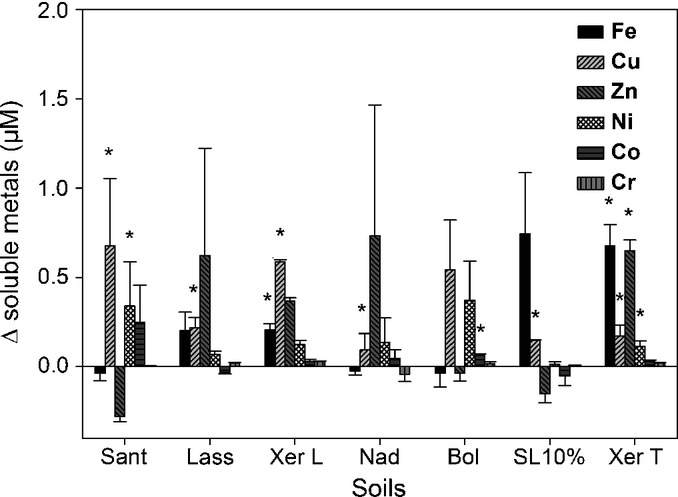
Differences (Δ) in soluble metal concentrations between the sampled bulk and rhizosphere soil solution calculated by subtracting metal rhizosphere from average bulk soil concentrations, averaged across both sampling events. Values represent means ± SE (*n* = 6). Statistically significant differences are highlighted: *, *P* < 0.05. Sant, Santomera; Xer T, Xeraco topsoil; Xer L, Xeraco subsoil; Bol, Bologna; Nad, Nadec; Lass, Lassee; SL 10%, Siebenlinden + 10% CaCO_3_.

2′-Deoxymugineic acid was identified in the rhizosphere soil solution of all seven experimental soils on both sampling events. However, DMA concentrations were not always above the limit of quantification in all three replicates (Table 4). Quantified DMA concentrations differed significantly between the soils, but also between the different sampling events, and ranged from 0.12 to 1.44 μM. Root growth differed between the soils, with particularly low biomass production in the saline soil (Nadec) (Table 3). The average change in soluble trace metals (Δ) as a result of the presence of wheat calculated for each metal separately (rhizosphere minus bulk soil solution concentrations) is shown in Fig. 3. Significantly enhanced Fe concentrations were detected in the rhizosphere of only two soils (Xeraco L, Xeraco T). On the other hand, soluble Cu was significantly increased in all soils but one (Bologna). Nickel, Zn, and Co concentrations were each significantly enhanced in only one of the seven soils (Ni, Santomera; Zn, Xeraco T; Co, Bologna). No statistically significant changes in soluble Cr were found.

**Table 4:** 2′-Deoxymugineic acid (DMA) concentrations (μM) in the rhizosphere soil solution of wheat (*Triticum aestivum* cv Tamaro) grown in different calcareous soils (Expt 2b)

		DMA (μM)	
		
Soil	Replicate	42 DAG	54 DAG
Santomera	1	0.32	0.77
	2	0.36	0.81
	3	0.52	1.44
Lassee	1	0.66	< LOQ
	2	0.43	0.13
	3	< LOQ	< LOQ
Xeraco L	1	0.78	0.31
	2	0.23	< LOQ
	3	0.41	< LOQ
Nadec	1	0.33	0.43
	2	0.14	< LOQ
	3	0.15	0.39
Bologna	1	< LOQ	< LOQ
	2	< LOQ	0.15
	3	0.20	0.20
SL 10%	1	1.04	0.24
	2	< LOQ	0.15
	3	0.62	0.34
Xeraco T	1	< LOQ	< LOQ
	2	0.26	0.19
	3	0.16	< LOQ

Samples were obtained by microsuction cups at 42 and 54 d after germination (DAG), limit of quantification (LOQ) < 0.1 μM, relative standard deviation (RSD) 5.8%.

## Discussion

### Repetitive and nondestructive sampling of DMA exudation rates from soil-grown wheat

Using a rhizobox setup combined with the REC system (Fig. S1), we were able to nondestructively and repeatedly sample unaltered DMA release from wheat grown on a soil with low Fe availability. Marschner *et al*. (1987) used an agar sheet to sample PS release from soil-grown plants on a single sampling occasion, but this technique did only allow intra-experimental comparisons, as the sampled root biomass cannot be accurately determined (not all roots will grow on the surface of the soil block) and the PS capture efficiency of the agar sheet was not considered. Consequently, the data presented in their study cannot be used for comparison with results presented here.

Repeated root exudate sampling by the REC tool revealed a continuous decrease in DMA release (g^–1^ root biomass) over time, with a particularly sharp drop in exudation rates observed between 33 and 35 DAG. DMA release thereafter ranged only between 13 and 5% of the release observed at the first sampling time point (21 DAG) (Fig. 1a). In nutrient solution culture studies, the same trend was observed; however, it was already occurring at a much earlier stage (< 20 DAG e.g. Clark *et al*., 1988; Zuchi *et al*., 2011). As most of these studies were carried out under zero Fe supply, we suggest that the early drop in PS release in nutrient solution culture could be attributed to impaired photosynthetic activity resulting from severe Fe deficiency. Results presented by Crowley *et al*. (2002), however, showed that, depending on barley cultivar and Fe supply, PS exudation rates can increase as well as decrease or remain constant with time (20 d of plant growth). This suggests that changes in PS exudation rates of plants grown under limited (but not zero) Fe supply depend on species/cultivar-specific plant internal Fe-use efficiency and that, depending on uptake efficiency as well as external Fe availability, Fe deficiency might also be overcome, leading to a decrease in PS production and release. Furthermore, timing and magnitude of the onset of PS release will also be governed by the Fe content of the seeds, with Fe reserves of wheat seeds being depleted *c*. 2 wk after germination (White & Veneklaas, 2012). In this study (Expt 1a), the decline in the SPAD value observed 13 DAG is likely to mark the time point of Fe seed reserve depletion. Despite the significant correlation between SPAD and DMA release, these results have to be considered with care as only few data points could be used in the statistical analysis.

By fitting a modified single exponential decay equation to the experimentally determined exudation data, we estimated the total amount of DMA released (21–47 DAG, Fig. S3) and compared it with Fe accumulated in the plant tissue (Table 5). These calculations suggest that, during the entire investigated period, about twice as much DMA is released than Fe taken up. However, further subdivision into shorter time intervals indicates that C investment into Fe acquisition was high (10-fold higher DMA release than Fe uptake) in the first 12 d of investigation, with first measurements being done 1 wk after Fe seed reserves were likely to be depleted. Thereafter, the measured DMA release did not exceed Fe uptake (Table 5). Despite the reasonably good fit of the selected function (*R* ² = 0.93), results from the data interpolation have to be interpreted with care because of the inhomogeneous distribution of measurements over time. Nevertheless, these rough estimates offer a valuable insight into the C investment strategy of wheat grown on Fe-deficient soil. The finding of a rather low excess in PS release compared with Fe accumulation is particularly surprising, as both adsorption to the soil matrix and microbial degradation have been found to decrease the Fe-solubilizing efficiency of PSs (Von Wiren *et al*., 1993b; Reichard *et al*., 2005). However, Fe might be additionally taken up by a PS-independent transport system (strategy I). There is genetic (Charlson & Shoemaker, 2006) and experimental evidence (Von Wiren *et al*., 1994) that strategy I features are still intact in grasses but are not up-regulated upon Fe deficiency (Von Wiren *et al*., 1993a). Considering the small contribution of DMA-C to total C analyzed (as DOC) in the root exudate samples, the role of other unidentified exudate compounds in solubilizing Fe from soil minerals might also have been underestimated. Snatching Fe from already soluble Fe–DOC complexes might be an efficient mechanism of Fe acquisition by PSs that has not yet been considered. Future work should assess whether PSs act as seek-and-fetch molecules, as has been supposed so far, or whether – at much lower exudation rates and concentrations – they only serve as transporters through the membrane, with other organic exudates being responsible for mobilizing the Fe in the rhizosphere.

**Table 5:** Plant (wheat, *Triticum aestivum* cv Tamaro) iron (Fe) accumulation and 2′-deoxymugineic acid (DMA) release over time

	Relative Fe accumulated*	Total Fe accumulated†	Time period	Δ Fe accumulated	Δ DMA released
					
DAG	% of harvest	μmol	DAG	μmol	μmol
21	50	0.3	**21–47**	**2.5**	**5.5**
33	53	0.6	21–33	0.3	3.4
35	54	1.1	33–35	0.5	0.5
41	74	2.1	35–41	1.0	1.1
47	100	2.8	41–47	0.7	0.5

Comparison of total DMA release and plant Fe accumulation during the experimental period (21–47 d after germination, DAG). Calculation of total DMA release was based on the root growth as well as the exudation model presented in Supporting Information Fig. S2 and S3, using only the 8 h peak release period as the results from the diurnal cycle sampling revealed negligible DMA release during the remaining 16 h. Bold numbers represent total accumulated Fe and DMA released over the entire investigated period.

* Relative Fe accumulation compared with the amount of Fe accumulated determined at the final harvest time point in the rhizobox root growth experiment (Expt 1b).

† Results from the final harvest (Expt 1a) corrected by the relative Fe accumulation observed in Expt 1(b).

As previously shown for nutrient solution-grown grasses (Takagi *et al*., 1984; Zhang *et al*., 1991; Ueno & Ma, 2009), we observed a distinct diurnal pattern, with the highest exudation rates of soil-grown wheat between 4 and 6 h after the onset of light at both sampling events, but with lower release rates at increased plant age (Fig. 2b). There is still an ongoing debate as to whether this exudation peak pattern is driven by light or temperature changes. Ueno & Ma (2009) and Ma *et al*. (2003) showed that colder temperatures (10°C) in the rhizosphere delayed the exudation peak and found no significant short-term shading effect (absence of light < 24 h). Reichman & Parker (2007), on the other hand, observed comparable peak patterns when wheat was grown either in a changing day : night temperature regime (13 : 23°C) or at constant temperature (23°C) and observed a significant drop in PS release when grown in the darkness for > 24 h. These results suggest that the PS peak release is affected, but not initiated, by changes in light intensity and/or temperature, yet governed by light exposure and consequently photosynthetic activity the day before. Even though glasshouse temperatures in this study did vary slightly depending on outside weather conditions, light period and intensity were kept constant. Therefore, changes in PS release as a result of light and temperature fluctuations can be ruled out.

A comparison of DMA release rates of soil-grown wheat (47 ± 11 pmol DMA g^−1^ s^−1^(mean ± SE, *n* = 5), 4 h average from 24 h stepwise sampling at 33 DAG; Fig. 2b) with rates observed in a previous study of the same cultivar grown in a nutrient solution with zero Fe supply (2200 ± 470 pmol DMA g^−1^ s^−1^ (*n* = 4), 4 h sampling, 28 DAG; (Schindlegger *et al*., 2014) shows that DMA release is *c*. 50 times lower in soil than in Fe-free nutrient solution culture. These results highlight the importance of investigating PS dynamics under natural soil conditions, as zero-Fe hydroponic growth conditions significantly overestimate PS release (see also Table 1).

Furthermore, the results from the 24 h stepwise sampling also show that, depending on the sampling time and duration, different exudation rates will be obtained (e.g. 4 h average (47 pmol g^−1^ s^−1^) vs 8 h average (32 pmol g^−1^ s^−1^), both 33 DAG). Therefore, extra care must be taken when comparing or extrapolating PS release rates, for example, for modeling purposes. Results from the 14 h stepwise exudate sampling showed that an 8 h sampling period is sufficient to capture *c*. 91% of total DMA released in 24 h. In general, we strongly recommend precise documentation of the sampling conditions (duration, daytime, plant age) as well as reporting exudation rates based on root DW using SI units (mol g^−1^ s^−1^), with the latter being particularly important to facilitate comparison across different studies.

### DMA release of wheat grown on different soils

Root exudate sampling (using a hydroponic setup after careful root washing) revealed that DMA and total DOC release rates of the same cultivar were significantly affected by the growth substrate (Fig. 2a,b) and negatively correlated with Zn shoot concentrations (Fig. 2, Tables 3, S2). Depending on whether the saline soil Nadec was included or excluded in the statistical test, significant correlations with Cu (negative, Nadec included) or Fe (positive, Nadec excluded) were also established, highlighting that the complexity of response reactions upon micronutrient deficiency (and salt stress) by soil-grown plants is still poorly understood. Both Zn and Cu deficiency have been found to trigger enhanced PS release (Zhang *et al*., 1991; Von Wiren *et al*., 1996; Gries *et al*., 1998); however, to the best of our knowledge, no data on the combined Cu and Zn deficiency have been reported so far. While some studies reported no effect of Zn deficiency on PS release (Gries *et al*., 1998; Chaignon *et al*., 2002), Rengel & Römheld (2000) observed that PS release by Zn-deficient bread and durum wheat cultivars was approximately five times lower and commenced at a later stage than was the case for Fe-deficient plants. Based on ^59^Fe tracer experiments, the authors further suggested that increased PS release related to Zn deficiency in wheat was triggered by reduced transport of Fe from roots to the shoots, with shoots responding to physiological deficiency of Fe by sending signals to increase exudation and transport up-regulation of PSs and PS–metal complexes. However, Suzuki *et al*. (2006) observed the opposite, namely a higher Fe translocation from roots to shoots in Zn deficient barley, suggesting a direct effect of Zn deficiency on PS release. These contradictive observations could be a result of species-specific differences in nutrient deficiency stress response and/or a result of different time points of the measurements. Rengel & Römheld (2000) only observed decreased Fe translocation in 13-d-old seeldlings of all tested cultivars, while older plants showed higher Fe transport from roots to shoots. Suzuki *et al*. (2006) only presented a single time point mesurement. Interestingly, very few studies exist on the effect of Cu deficiency on PS release. Gries *et al*. (1998) found that PS exudation was increased in Cu-deficient *Hordelymus europaeus* and followed the same diurnal release rhythm that can be found under Fe deficiency; however, release rates were 10 times lower than for Fe-starved plants. Gries *et al*. (1998) also stated that PS release under Cu deficiency occurred at an even earlier time point than under moderate Fe deficiency, though the data were not explicitly shown in the publication. Repeated sampling of DMA release over time (Expt 1a, Fig. 1) clearly showed that DMA exudation significantly differed depending on the sampling time point. Considering the soil-driven differences in plant micronutrient nutrition, not only DMA release but also the timing is likely to differ for the same cultivar when grown on different soils. Consequently, presented correlations of this one-time-point sampling should be interpreted with care. Clearly, more research is needed to elucidate PS dynamics under micronutrient deficiencies (Fe, Zn, and Cu), with particular focus on measurement timing.

Despite the lowest trace metal wheat tissue concentrations observed on the saline soil Nadec (Table 3), salinity could also have a strong effect on DMA release. Daneshbakhsh *et al*. (2013) found a two- to threefold increase in PS release rates of Zn-deficient wheat cultivars (calculated by converting exudation on a root DW basis and assuming constant root growth rates) when grown in the presence of 120 mM NaCl solution (corresponds to *c*. 12 mS cm^−1^ electric conductivity (EC)). The authors hypothesized that increased PS exudation rates under saline conditions were either an active salt stress response reaction as a result of the reduction of free Fe and Zn activity in the nutrient solution and/or a consequence of higher membrane permeability. In our study, DMA release was nine to 165 times higher in the saline (EC, 6.3 mS cm^−1^ in a soil saturation extract) than in the other soils investigated. In soil, salinity will not affect the free Fe activity as it is imposed by Fe(hydr)oxide phases present in the soil (Morel & Hering, 1993); however, free Zn and Cu activity might still be affected by high ionic strength. In any case, the higher contribution of DMA to total C released by wheat grown in the saline soil found in our study suggests actively induced PS formation and secretion rather than passive leakage resulting from a salinity-driven increase in membrane permeability.

2′-Deoxymugineic acid release rates of wheat grown on the highly calcareous Santomera soil sampled either hydroponically (pot Expt 2a) or nondestructively using the REC tool (rhizobox, Expt 2b) were in the same range (Expt 2a, 4 h sampling at 44 DAG, 2.2 ± 1.4 pmol g^−1^ s^−1^ (mean ± SE, *n* = 3, Fig. 3a) vs Expt 1(a), 24 h-sampling, 46 DAG, 5.1 ± 1.5 pmol g^−1^ s^−1^ (*n* = 5, calculated 4 h average from data presented in Fig. 2a)). In contrast to the strong overestimation of PS release in zero-Fe nutrient solution culture (Table 1), this suggests that after careful removal of the soil, hydroponic sampling after soil growth can still be used as simple tool to obtain realistic PS release rates under natural growth conditions.

### DMA and trace metal concentrations in the rhizosphere of wheat grown on different soils

*In situ* sampled DMA concentrations in the rhizosphere of wheat grown on different experimental soils ranged from below detection to 1.44 μM and differed between the soils as well as between the sampling events (Table 4). Considering the random positioning of the MSCs within the pots and continuous and nonrestricted root growth, no information on the distance between roots and the suction tube can be provided. Marschner *et al*. (1987) showed that root tips, in particular, release two to five times larger quantities of PS than basal roots. Observed differences in DMA concentrations between the two sampling events could therefore result from both changes in number and position of root tips in the vicinity of the MSC porous tube and changes in DMA release rates with time. Consequently, presented DMA concentrations across the three sampling events should be considered as the ‘rhizosphere average’.

Differences in DMA concentration between the soils could also be soil-dependent, as plant growth (root biomass and architecture) and DMA release rates are affected by soil chemical properties (particularly Fe phytoavailability, salinity); DMA adsorption and desorption dynamics as well as; microbial activity and DMA biodegradation.

Since microbial degradation was considered to significantly aggravate DMA recovery from the soil solution, the pots were watered with a Bronopol solution the day before the first and third sampling events. In a recent study, Rousk *et al*. (2010) showed that Bronopol almost completely inhibited bacterial but not fungal activity for 4 d. Therefore, it is uncertain whether DMA mineralization was completely or only partly inhibited by the addition of Bronopol, as this was not directly tested. However, it has been shown that bacteria dominate the microbial community under alkaline conditions (Rousk *et al*., 2009b), which is supported by our own, as yet unpublished, findings showing that, based on phospholipid fatty acid analysis (PLFA), fungi accounted for only *c*. 6% of the total amount of microbial biomarkers in this soil. We therefore consider observed differences in DMA soil solution concentration to be mainly driven by soil-dependent differences in DMA release, by soil-specific sorption of DMA and by changes in distance and number of root tips in the vicinity of the MSCs. Despite some methodological drawbacks, MSC sampling allows the investigation of geochemical ‘active’ concentrations of PS in the rhizosphere soil solution. Our results clearly demonstrate that solubilization efficiency studies using PS concentrations in a mM range do not reflect concentrations occurring under natural growth conditions.

Unexpectedly, Cu soil solution concentrations, in particular, were significantly increased by the presence of plants in all but one soil (Bologna), while significantly enhanced Fe, Ni, Zn and Co concentrations were only found occasionally (one or two soils out of seven) (Fig. 3). The absence of increased Fe solubility in the rhizosphere may be explained, in part, by the relatively high uptake rates of the Fe–PS complex related to the preferential uptake mechanism for this complex (Römheld & Marschner, 1990; Gries *et al*., 1995). It can therefore be expected that other unidentified DOC components also contributed to the observed increase in metal solubility, with Cu being known to show a particularly high affinity for soil organic matter (Oorts, 2013).

### Theoretical considerations – modeling unaltered DMA concentration in the rhizosphere

**Fig. 4:**
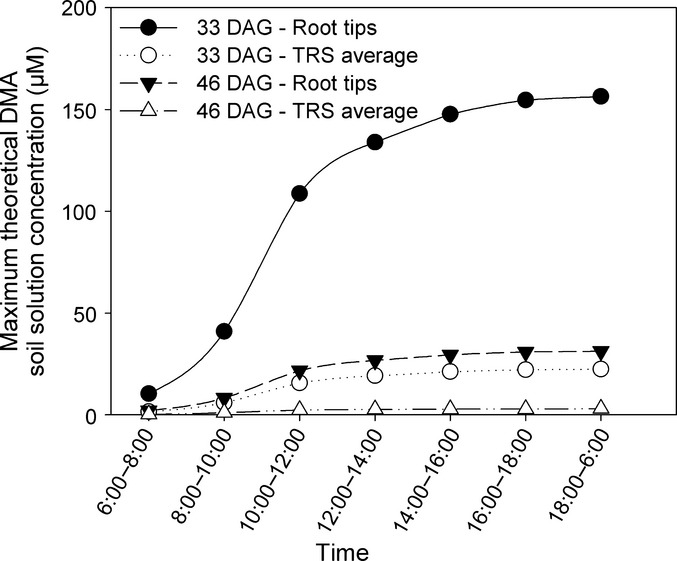
Maximum theoretical soil solution concentrations of 2′-deoxymugineic acid (DMA, μM) after 24 h in the close vicinity of wheat (*Triticum aestivum* cv Tamaro) roots (0.5 mm distance from the root surface) grown on the calcareous Santomera soil, calculated based on exudation rates obtained in the 24 h stepwise sampling at 33 and 46 d after germination (DAG) and assuming a uniform root diameter (0.5 mm). Closed symbols, theoretical DMA concentrations around root tips assuming hotspot exudation behavior (i.e. active proportion of root biomass is only 20%); open symbols, averaged theoretical DMA concentration across the total root system (TRS i.e. active proportion of root biomass is 100%).

Using experimentally determined DMA release rates, theoretical DMA rhizosphere soil solution concentrations were calculated according to Römheld (1991), assuming unaltered DMA release (no adsorption, no reuptake, no microbial degradation), a uniform root diameter of 0.5 mm, and a thickness of the surrounding rhizosphere cylinder of 0.5 mm (Table 6). However, in contrast to Römheld (1991), we do not consider the extrapolation of DMA exudation rates obtained during the 4 h of peak release to daily release rates to be valid, as this will lead to an overestimation of DMA soil solution concentration (as discussed earlier). Based on exudation rates obtained from barley grown in zero-Fe hydroponic culture and sampled for 4 h during PS peak release, Römheld (1991) concluded that unaltered DMA rhizosphere concentrations up to 3000 μM can be expected. Recalculating these results by considering the diurnal peak release dynamics, we suggest that maximum rhizosphere concentrations after 4 h of DMA peak release are only in the range of 500 μM. However, as demonstrated in this study, nutrient solution culture experiments significantly overestimate PS release rates (factor of 50 for wheat). Using the data from the pot experiment (Expt 2a) in this study, our calculations reveal that the theoretical maximum unaltered DMA concentrations (no microbial degradation and sorption considered) after the peak release period in soil surrounding exudation hotspots like root tips will range from 0.6 to 9 μM in the saline soil, even up to 85 μM DMA (Table 6). Calculating a rhizosphere average (assuming uniform DMA release across the entire root system), maximum theoretical concentrations will range between 0.1 and 2 μM (17 μM for the saline soil), which is in the same range as our experimental observations (Table 4). The large discrepancy between experimental results and calculated concentrations in the saline soil could be partly explained by the low root density of wheat in the pot (Table 3) and consequently also around the MSC, leading to a stronger dilution of DMA in these samples. Considering the diurnal PS release dynamics, the maximum theoretical soil solution concentrations of DMA (μM) in the close vicinity of wheat roots (0.5 mm) in the course of the day were calculated based on exudation rates obtained in the 24 h stepwise sampling at 33 and 46 DAG (Fig. 4). Again assuming unaltered PS hotspot release mainly via root tips, theoretical maximum concentrations (i.e. input only) of DMA were 156 and 31 μM (33 and 46 DAG, respectively), while, averaged across the entire root system, the maximum rhizosphere DMA concentrations were 22 and 3 μM respectively.

**Table 6:** Maximum theoretical 2′-deoxymugineic acid (DMA) concentrations (no microbial degradation or adsorption) in the close vicinity of wheat roots (*Triticum aestivum* cv Tamaro; 0.5 mm distance from the root) grown in different soils compared with modified calculations published by Römheld (1991) using nutrient solution (zero iron (Fe)) grown barley as the model plant

		Barley*	Wheat (*Triticum aestivum* cv Tamaro)						
			
Parameter	Units	Zero Fe solution	Sant	Bol	Xer L	Xer T	Lass	Nadec	SL+10%
Root mass	g	0.03*	1.4	1.7	3.4	1.9	2.2	0.8	1.7
Root density	g mm^−3^	5.8 e^−5^*	5.8 e^−5^*	5.8 e^−5^*	5.8 e^−5^*	5.8 e^−5^*	5.8 e^−5^*	5.8 e^−5^*	5.8 e^−5^*
Root diameter	mm	0.5*	*0.5*	*0.5*	*0.5*	*0.5*	*0.5*	*0.5*	*0.5*
Root + rhizosphere cylinder diameter	mm	1.5*	*1.5*	*1.5*	*1.5*	*1.5*	*1.5*	*1.5*	*1.5*
Total root length	mm	2325*	124 522	145 631	296 876	168 817	191 943	67 860	144 988
Root volume	mm³	465*	24 450	28 595	58 291	33 147	37 688	13 324	28 468
DMA release rate	μmol per plant per 4 h	0.090†	0.044	0.007	0.012	0.010	0.010	0.454	0.103
Volume of the rhizosphere cylinder	mm³	725*	18 710	20 198	45 430	20 657	23 128	7789	38 339
Volume of water in rhizosphere cylinder (WC 25%)	mm³	181*	4678	5050	11357	5164	5782	1947	9585
**DMA concentration in the rhizosphere cylinder:**									
Active root zones (20% of total) – root tips	μM	493†	4.5	0.6	0.5	0.7	0.7	85	9.1
Total root system (TRS) average	μM	99†	0.9	0.1	0.1	0.1	0.1	17	1.8

No microbial degradation or adsorption was considered. Italic numbers mark the assumption made for root and rhizosphere dimensions.

* Values from Römheld (1991).

† In contrast to extrapolated daily rates used by Römheld (1991), the release period was reduced to 4 h during which DMA samples were taken and DMA peak release was observed. Extrapolation of release rates on a daily basis using DMA exudation observed during peak release will lead to an overestimation of DMA concentrations in the rhizosphere.

Sant, Santomera; Bol, Bologna; Xer T, Xeraco topsoil; Xer L, Xeraco subsoil; Lass, Lassee; Nad, Nadec; SL + 10%, Siebenlinden + 10% CaCO_3_.

Besides offering a valuable insight into the maximum unaltered PS concentrations in the rhizosphere under natural growth conditions, these calculations further highlight the importance of investigating PS release dynamics in soil. Experimental results and theoretical considerations in this study demonstrate that extra care must be taken when using and extrapolating results obtained from hydroponic studies for model simulations. Modeling is a powerful tool to elucidate rhizosphere processes and underlying mechanisms. More complex mathematical models are needed to include relevant processes like microbial degradation and sorption, as well as trace metal solubilization, complexation and uptake, to fully capture PS dynamics in soil.

### Conclusion

Results of this study demonstrate that the C investment in PS synthesis and release of wheat grown on soils with low Fe availability is significantly smaller than previously suggested, as we found that zero-Fe hydroponic culture overestimated PS release rates (normalized by root DW) by a factor of *c*. 50. Additionally we observed that, when grown on a calcareous soil, the C investment (i.e. total PS release) of wheat in Fe acquisition was higher within the first 3 wk after Fe seed reserves were depleted, and declined significantly thereafter. Investigating DMA exudation of the same wheat cultivar grown on different calcareous soils revealed complex interactions between the nutritional status of the plant (affected by soil characteristics, particularly trace metal availability and salinity) and the related regulation (amount and timing) of PS release. While strictly controllable growth conditions (i.e. nutrient solution culture) facilitate the identification and functional attribution of trace metal deficiency-driven plant response mechanisms, our results clearly show that investigating PS release dynamics under natural growth conditions is an indispensable prerequisite for better understanding the relevant mechanisms involved in plant Fe (and other trace metal) nutrition in soil.

**About New Phytologist**

- *New Phytologist* is an electronic (online-only) journal owned by the New Phytologist Trust, a **not-for-profit organization** dedicated to the promotion of plant science, facilitating projects from symposia to free access for our Tansley reviews.
- Regular papers, Letters, Research reviews, Rapid reports and both Modelling/Theory and Methods papers are encouraged. We are committed to rapid processing, from online submission through to publication ‘as ready’ via *Early View* - our average time to decision is <25 days. There are **no page or colour charges** and a PDF version will be provided for each article.
- The journal is available online at Wiley Online Library. Visit http://www.newphytologist.com to search the articles and register for table of contents email alerts.
- If you have any questions, do get in touch with Central Office (np-centraloffice@lancaster.ac.uk) or, if it is more convenient, our USA Office (np-usaoffice@ornl.gov)
- For submission instructions, subscription and all the latest information visit http://www.newphytologist.com

## Supporting Information

**Fig. S1** Schematic drawing of the rhizobox-REC assembly. **Fig. S2** Root biomass development of wheat (*Triticum aestivum* cv Tamaro) grown in rhizoboxes over time. **Fig. S3** Curve fitting to the experimentally determined DMA exudation data. **Table S1** Significant differences in SPAD readings and correlation with DMA release rates **Table S2** Correlation analysis of investigated plant and soil parameters
