# Supplementary material for: Root exudation of phytosiderophores from soil-grown wheat
Source: New Phytol. 2014 Jun 2;203(4):1161–74. doi: 10.1111/nph.12868 (PMC4143957; doi:10.1111/nph.12868)
Supplement: Supplementary file 1 [file nph0203-1161-SD1.docx]

**Supporting Information: Figure S1-S3 and Table S1-S2**

Figure S1 Schematic drawing of the rhizobox-root exudate collector assembly allowing non-destructive and repetitive collection of unaltered root exudates (modified according to **Oburger E, Dell'mour M, Hann S, Wieshammer G, Puschenreiter M, Wenzel WW. 2013.** Evaluation of a novel tool for sampling root exudates from soil-grown plants compared to conventional techniques. *Environmental and Experimental Botany* **87**: 235-247.)

Figure S2 Root biomass development of wheat (*Triticum aestivum* cv Tamaro) grown in rhizoboxes on a highly calcareous soil (experiment 1b) expressed as percentage of the root biomass measured at the final sampling time point (47 DAG). The linear equation was used to correct for changes in root biomass over time required for calculating exudation rates sampled with the root exudate collecting tool (experiment 1a)

Figure S3 Curve fitting to the experimentally determined DMA exudation data (experiment 1a). Curve fitting was carried out using SigmaPlot 12.5 Systat Software Inc.

Table S1 a) Significant differences in SPAD readings during the experimental period determined by means of ANOVA including the *Student-Newman-Keuls (SNK)* post hoc test with p < 0.05. b) Correlation analysis between DMA release rates (DMA ex rate) and SPAD readings. Due to the difference in number of measurements only the averages for each exudate sampling time point were used.

| ANOVA, *SNK* (*p* < 0.05) | | | | |
| --- | --- | --- | --- | --- |
| DAG 7 |  | b | c |  |
| 10 |  | b | c |  |
| 13 | a | b | c |  |
| 15 | a |  |  |  |
| 16 | a | b |  |  |
| 19 | a | b | c |  |
| 21 | a | b |  |  |
| 26 | a | b | c |  |
| 31 | a | b | c |  |
| 33 | a | b | c |  |
| 35 | a | b | c |  |
| 38 |  |  | c |  |
| 41 |  | b | c |  |
| 47 | a | b | c |  |

a)

b)

| **Correlation** | | |
| --- | --- | --- |
|  |  | SPAD |
| DMA ex rate | *Pearson c.* | -0.941 |
|  | *p* | 0.005 |

Table S2 Correlation analysis of investigated plant and soil parameters including exudation rates of DMA (DMA exrate), total C (Ctot exrate), and shoot tissue concentrations for Fe, Zn and Cu of wheat grown on 7 different calcareous soils, as well as soil chemical parameters: DTPA extractable Fe, Zn, Cu and electric conductivity measured in a 1:10 water extraction as measure of salinity a) Correlation analysis including all 7 experimental soils and b) excluding the saline soil Nadec.

| 1. **Correlation analysis including all 7 experimental soils** | | | | | |
| --- | --- | --- | --- | --- | --- |
|  | |  |  |  |  |
|  | | DMAexrate | ShootFe | ShootZn | ShootCu |
| DMAexrate | *Pearson c.* |  | -0.380 | **-0.693** | **-0.590** |
|  | *p* |  | 0.089 | **0.000** | **0.005** |
| Shoot Fe | *Pearson c.* | -0.380 |  |  |  |
|  | *p* | 0.090 |  |  |  |
| ShootZn | *Pearson c.* | **-0.693** |  |  |  |
|  | *p* | **0.000** |  |  |  |
| ShootCu | *Pearson c.* | **-0.590** |  |  |  |
|  | *p* | **0.005** |  |  |  |
| DTPAFe | *Pearson c.* | -0.184 | -0.285 |  |  |
|  | *p* | 0.425 | 0.211 |  |  |
| DTPACu | *Pearson c.* | **-0.496** |  |  | 0.320 |
|  | *p* | **0.020** |  |  | 0.158 |
| DTPAZn | *Pearson c.* | -0.293 |  | **0.666** |  |
|  | *p* | 0.200 |  | **0.001** |  |
| Ctot exrate | *Pearson c.* | 0.329 | 0.244 | 0-.355 | 0.136 |
|  | *p* | 0.150 | 0.286 | 0.114 | 0.557 |
| Salinity (EC) | *Pearson c.* | **.944** | **-0.543** | **-0.579** | **-0.635** |
|  | *p* | **0.00** | **0.011** | **0.006** | **0.002** |

| **b) Correlation analysis excluding the saline Nadec soil** | | | | | |
| --- | --- | --- | --- | --- | --- |
|  |  | DMAexrate | ShootFe | ShootZn | ShootCu |
| DMAexrate | *Pearson c.* | 1 | **0.641** | **-0.612** | -0.020 |
|  | *p* |  | **0.004** | **0.007** | 0.936 |
| ShootFe | *Pearson c.* | **0.641** |  |  |  |
|  | *p* | **0.004** |  |  |  |
| ShootZn | *Pearson c.* | **-0.612** |  |  |  |
|  | *p* | **0.007** |  |  |  |
| ShootCu | *Pearson c.* | -0.020 |  |  |  |
|  | *p* | 0.936 |  |  |  |
| DTPAFe | *Pearson c.* | -0.133 | -0.419 |  |  |
|  | *p* | 0.599 | 0.083 |  |  |
| DTPAZn | *Pearson c.* | -0.395 |  | **0.685** | -0.034 |
|  | *p* | 0.105 |  | **0.002** | 0.895 |
| DTPACu | *Pearson c.* | **-0.553** |  |  | 0.065 |
|  | *p* | **0.017** |  |  | 0.797 |
| Ctot exrate | *Pearson c.* | 0.461 | 0.448 | -0.225 | 0.409 |
|  | *p* | 0.054 | 0.062 | 0.370 | 0.092 |
| Salinity (EC) | *Pearson c.* | -0.161 | -.438 | 0.327 | -0.251 |
|  | *p* | 0.523 | 0.069 | 0.185 | 0.314 |
